# Characterization of Endothelial and Smooth Muscle Cells From Different Canine Vessels

**DOI:** 10.3389/fphys.2019.00101

**Published:** 2019-02-12

**Authors:** Loes A. Oosterhoff, Hedwig S. Kruitwagen, Monique E. van Wolferen, Bas W.M. van Balkom, Michal Mokry, Nico Lansu, Noortje A.M. van den Dungen, Louis C. Penning, Talitha C.F. Spanjersberg, Johannes W. de Graaf, Tomas Veenendaal, Flin Zomerdijk, Joost O. Fledderus, Bart Spee, Frank G. van Steenbeek

**Affiliations:** ^1^Department of Clinical Sciences of Companion Animals, Faculty of Veterinary Medicine, Utrecht University, Utrecht, Netherlands; ^2^Nephrology and Hypertension, Division of Internal Medicine and Dermatology, University Medical Center Utrecht, Utrecht, Netherlands; ^3^Division of Pediatrics, Wilhelmina Children’s Hospital, University Medical Center Utrecht, Utrecht, Netherlands; ^4^Epigenomics Facility, University Medical Center Utrecht, Utrecht, Netherlands; ^5^Department of Cardiology, Division of Heart and Lungs, University Medical Center Utrecht, Utrecht, Netherlands

**Keywords:** angiogenesis, cell model system, endothelial cells, vascular cell interaction, vascular smooth muscle cells

## Abstract

Vasculature performs a critical function in tissue homeostasis, supply of oxygen and nutrients, and the removal of metabolic waste products. Vascular problems are implicated in a large variety of pathologies and accurate *in vitro* models resembling native vasculature are of great importance. Unfortunately, existing *in vitro* models do not sufficiently reflect their *in vivo* counterpart. The complexity of vasculature requires the examination of multiple cell types including endothelial cells (ECs) and vascular smooth muscle cells (VSMCs), as well as vessel location in the body from which they originate. The use of canine blood vessels provides a way to study vasculature with similar vessel size and physiology compared to human vasculature. We report an isolation procedure that provides the possibility to isolate both the endothelial and smooth muscle cells from the same vessels simultaneously, enabling new opportunities in investigating vasculature behavior. Canine primary ECs and VSMCs were isolated from the vena cava, vena porta and aorta. All tissue sources were derived from three donors for accurate comparison and to reduce inter-animal variation. The isolation and purification of the two distinct cell types was confirmed by morphology, gene- and protein-expression and function. As both cell types can be derived from the same vessel, this approach allows accurate modeling of vascular diseases and can also be used more widely, for example, in vascular bioreactors and tissue engineering designs. Additionally, we identified several new genes that were highly expressed in canine ECs, which may become candidate genes for novel EC markers. In addition, we observed transcriptional and functional differences between arterial- and venous-derived endothelium. Further exploration of the transcriptome and physiology of arteriovenous differentiation of primary cells may have important implications for a better understanding of the fundamental behavior of the vasculature and pathogenesis of vascular disease.

## Introduction

Two essential cell types in blood vessels are endothelial cells (ECs), which line the inside of all blood vessels, and vascular smooth muscle cells (VSMCs), which regulate vessel stability. Although the main function of ECs is to provide a barrier between the blood and the rest of the body, they also maintain vascular homeostasis by orchestrating the selective exchange of nutrients, oxygen and immune cells between tissues or organs, and act as key players in angiogenesis and vasculogenesis, aiding the formation of new microvasculature to create an organized network (Jain, 2003). Human umbilical vein ECs (HUVECs) and endothelial progenitor cells (EPCs) are widely used to model ECs *in vitro*. Isolation of these two cell types does not require surgery or even death of the donor, as HUVECs are derived from the umbilical cord and EPCs are derived from blood samples (Melero-Martin et al., 2007, 2008; Emontzpohl et al., 2017). Vascular behavior of HUVECs is the “gold standard” in vascular research (Sacks et al., 1978); they have been well studied and have provided vital information about the role of ECs in maintaining homeostasis and to assess risk factors of vascular disease (Gonzalez-Miguel et al., 2015; Duan et al., 2016; Yang et al., 2016). Unfortunately, using HUVECs and EPCs to model adult blood vessels *in vitro* remain challenging due to molecular and functional differences between ECs (Hauser et al., 2017).

The extracellular matrix (ECM) is essential for both vasculogenesis (*de novo* formation of blood vessels) and angiogenesis (the formation of blood vessels from pre-existing vessels). The ECM is diverse and dynamic, and placement and conformation of its components dictate its overall physiological properties and influence the behavior of neighboring cells (Jain, 2003; Zhu et al., 2013). Blood vessel formation also requires the support of mural cells, such as VSMCs, pericytes, and a mixture of macrophages, fibroblasts, and dendritic cells, which contribute to ECM production and structure of the new vasculature (Michiels, 2003; Halper, 2018). Additionally, surrounding VSMCs release growth factors such as vascular endothelial growth factor (VEGF), which triggers ECs in response to initiate angiogenesis (Korff et al., 2001). Culture systems often consist of mono-layered ECs without the support of the naturally surrounding cells and ECM (Edmondson et al., 2014). To increase culture complexity, HUVECs are often used in co-culture models with mesenchymal cells or fibroblasts, which are known for their production of ECM components (Newman et al., 2011; Pill et al., 2015). However, these cells do not naturally interact with ECs in the umbilical cord and therefore do not accurately represent blood vessel physiology (Zhang et al., 2012; Cheung et al., 2015; Strassburg et al., 2016).

Efforts have been made to isolate primary ECs and VSMCs from different vessels and use them for *in vitro* vascular models (Ganesan et al., 2017); however, a drawback of the procedure is that the cells are derived from two different tissue sources. It has been suggested that EC characteristics, regardless of whether they originate from arteries or veins, differ only in morphology due to hemodynamic pressure (Ives et al., 1986); therefore, single EC lines were commonly used for various vascular research questions. More recently, however, it has been reported that the morphology and functionality of ECs do indeed depend on their originating vessel and differ with respect to genetic background and micro-environmental factors (Aranguren et al., 2013; Hauser et al., 2017; Kutikhin et al., 2018). Obtaining human donor material from adult vessels is a challenge, which emphasizes the urge of an animal model capable of bridging this gap.

The canine is a large animal model that resembles human vasculature closely with respect to vessel size. To study the *in vivo* interactions between vascular cells, we isolated and characterized primary ECs and VSMCs from the same vessels in a canine model. We investigated whether this new procedure produced viable cells for a blood vessel model will aid in the translation toward the human physiology of adult vasculature. Both primary ECs and VSMCs from the same vessel were molecularly and functionally characterized and present a novel model for *in vitro* vasculogenesis research. Moreover, these two cell types could provide a strong base for transplantation purposes. The emergence of precise three-dimensional (3D) models and tissue engineering (TE) highlight the importance of discriminating between specific cell types and donor variations (Kim et al., 2017). The procedure we describe allows (i) the isolation of ECs and VSMCs from different vessel locations, (ii) the direct isolation of ECs and VSMCs from the same vessel, and (iii) the establishment of transplantation of vascularized 3D engineered tissue in a large animal model.

## Materials and Methods

### Ethics Statement

For this study, blood vessels from healthy male dogs (*n* = 3; age 12–14 months, with an average of 26 kg) were used. The vessels were collected as surplus material from fresh canine cadavers used in unrelated research on pacemakers (Sprenkeler et al., 2018) (Utrecht University 3R policy). Individuals showed no sign of heart failure at time of euthanasia.

### Culture

#### Isolation of Primary ECs and VSMCs

Endothelial cells and VSMCs in this study originated from the abdominal section of the vena cava, the vena porta, and the aorta of three fresh canine cadavers. Adhering (fatty and connective) tissue was carefully removed with surgical scissors and any branches of the vessel were closed with ligatures. Inversion of the blood vessel was accomplished by clamping a curved Halsted mosquito forceps on one vessel end from the inside, followed by slow retraction causing the vessel to invert. Both vessel ends were closed with purse-string sutures (4-0 Vicryl, Ethicon, Cincinnati, OH, United States), and the vessel was then rinsed in Hank’s Balanced Salt Solution (Life Technologies, Carlsbad, MA, United States). The EC layer was digested for 30 min in collagenase type II (0.15 U/mL, Life Technologies) and dispase (0.15 U/mL, Life Technologies) in Dulbecco’s Modified Eagle’s Medium (DMEM) GlutaMAX (Life Technologies) at 37°C. Canine primary ECs (CaPECs) were obtained by centrifugation at 250 × *g* for 5 min. After CaPEC isolation, the vessel was minced into small pieces and a 4-h digestion with collagenase type II (0.09 U/mL) in DMEM GlutaMAX was performed at 37°C, allowing collection of the VSMC fraction. Large pieces of the tissue were removed by a 70 μM strainer prior to centrifugation at 250 × *g* for 5 min at room temperature (RT).

#### Culture Conditions

Canine primary ECs were cultured on 0.1% gelatin (Sigma–Aldrich, Saint Louis, MO, United States) pre-coated culture plates, coated for 30 min at 37°C, in Canine Endothelial Cells Growth Medium (CECGM, Cell Applications, San Diego, CA, United States). VSMCs were cultured in DMEM GlutaMAX, 10% Fetal Calf Serum (FCS, Life Technologies) and 100 μg/mL Primocin (Invivogen, Toulouse, France). Cultures were incubated in a humidified incubator at 37°C with 5% CO_2_ in air.

### Characterization

#### Immunocytochemistry

To characterize and assess the purity of the obtained cell populations, immunocytochemistry was performed. Cells were cultured for 24–48 h on chamber slides (Millipore, Burlington, MA, United States) at a concentration of 20,000 cells/cm^2^ under the described culture conditions until a 90% confluence was obtained. Cells were fixed for 4 min in ice-cold acetone/methanol (1:1 v/v) and air dried for 10 min at RT. Slides were washed with phosphate buffered saline (PBS) with 0.1% Tween 20 (Sigma) and blocked with 10% goat serum in PBS prior to incubation with rabbit primary antibody against CD31 (Abcam, Cambridge, United Kingdom), Calponin (Abcam), vWF (Dako, Santa Clara, CA, United States), and α-SMA (Alexa Fluor 488 labeled, Abcam) overnight at 4°C. Anti-rabbit IgG (DAKO, Agilent, Santa Clara, CA, United States) was used as isotype control. Incubation of secondary Alexa Fluor 488 labeled goat-anti-rabbit (Dako) was performed for 1 h at RT for the CD31, Calponin, α-SMA, and isotype conditions. Counterstaining was performed with 1 μg/mL DAPI (Sigma) for 10 min at RT.

#### Whole Transcriptome Sequencing

Total RNA was isolated from CaPECs and VSMCs isolated from the vena cava, the vena porta, and the aorta (all in passage 3) from healthy dogs (*n* = 3), using a RNeasy Mini Kit (Qiagen, Venlo, Netherlands) and residual DNA was removed with an on-column DNase digestion. The polyadenylated mRNA fraction was isolated using Poly(A) Beads (NEXTflex, Bioo Scientific, Austin, TX, United States). Sequencing libraries were prepared using the Rapid Directional RNA-Seq Kit (NEXTflex) and sequenced on Illumina NextSeq500 to produce single-end 75 base long reads. RNA-seq reads were aligned to the reference genome canFam3 using STAR version 2.4.2a (Dobin et al., 2013). Read groups were added to the BAM files with Picard’s AddOrReplaceReadGroups (v1.98). The BAM files are sorted with Sambamba v0.4.5 and transcript abundances were quantified with HTSeq-count version 0.6.1p1 (Anders et al., 2015), using the union mode. Subsequently, reads per kilobase of transcript per million reads sequenced (RPKMs) were calculated with edgeR’s rpkm() function (Robinson et al., 2010). The raw and analyzed files have been uploaded to Gene Expression Omnibus under the accession GSE118029.

ToppFun was used for functional enrichment analysis based on functional annotations and protein interactions networks (Chen et al., 2009).

#### 3D Spheroid Assay

A 3D spheroid model was applied to study the sprouting capability of all isolated cells. For this model, 0.5 g of dextran micro carrier beads (Cytodex 3, GE Healthcare, Eindhoven, Netherlands) were prepared in a final volume of 25 mL PBS to reach approximately 60,000 beads/mL. A cell suspension of 200,000 cells/350 μl growth media with 1% v/v Glutamax (Life Technologies) was added to 100 μl of autoclaved micro carriers. The cell and bead suspension was incubated for 4 h at 37°C with intermittent gentle agitation. After incubation, the cell-bead mixture was incubated on a 0.1% gelatin pre-coated surface overnight at 37°C in a humidified incubator, encouraging unattached cells to adhere to the coating and therefore be removed from the suspension.

Per test condition, Matrigel (Corning, Tewksbury, MA, United States) was mixed with culture medium in a 2:1 ratio. Matrigel mixture (100 μl) was spread evenly over the surface of a Cellstar flat bottom 48-well plate (Greiner, Frickenhausen, Germany). Gel was solidified for 30 min at 37°C. The cell-bead suspension was pelleted and medium replaced with a mixture of 100 μl growth medium and 200 μl Matrigel, and 60 μl was seeded on top of the solidified Matrigel. After solidifying for 30 min at 37°C, 500 μl per well of growth media was added and culture was continued in a humidified atmosphere at 37°C with 5% CO_2_ in air. Pictures were taken after 7 days (Olympus CKX41).

#### Tube Formation Assay

Tube formation was assessed using micro-slides (Ibidi, Martinsried, Germany). Per well 10 μl Matrigel was added and solidified for 30 min at 37°C, prior to seeding 1.0^∗^10^4^ cells per well in 50 μl CECGM. Cells were imaged 6 h after seeding (Olympus CKX41).

#### Contraction Assay

A hydrogel disc model was used to investigate differences in contractile capacity between CaPECs and VSMCs (Manetti et al., 2017). Collagen type I (Sigma) was diluted to 3 mg/mL with Milli-Q H2O (Millipore) and brought to a pH of 7.4 with NaOH (Merck, Amsterdam, Netherlands). Of both the VSMCs and CaPECs, a cell suspension of 300,000 cells/mL in VSMC culture medium was diluted 1:2 with the collagen, and distributed as 500 μl per well [flat bottom 24-well culture plate with cell repellent surface (Greiner)]. Gelation was initiated by incubation for 1 h at 37°C, after which culture medium was added. After 48 h, the discs were imaged using a Gel Doc 2000 (Bio-Rad, Veenendaal, Netherlands). The RGB data of the images were converted to a 2-bit black/white image using ImageJ software, allowing measurements of the relative square area of the discs.

#### Statistics

Immunocytochemistry, 3D spheroid assay, tube formation assay, and contraction assay were all performed using triplicates. Differentially expressed genes in the whole transcriptome data were identified using the DESeq2 package (Love et al., 2014) with standard settings. A heatmap was generated using the R-package gplots. Genes with log2 fold change larger than 0.4 and a *p*-value < 0.05 were considered differentially expressed. Relative square areas of the discs were tested for normal distribution using the Shapiro–Wilk normality test (*p* = 0.0076). Significant differences in the contraction assay were determined by performing a Wilcoxon signed rank test.

## Results

### Culture Characteristics

**FIGURE 1:**
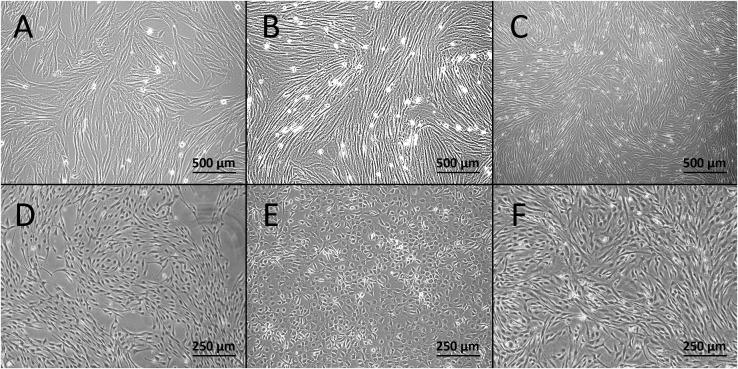
Light microscopy images of the derived cells at passage 1. Confluent cultures of VSMCs derived from **(A)** vena cava, **(B)** vena porta, and **(C)** aorta. Cultures of canine primary endothelial cells (CaPECs) originating from equivalent vessels: **(D)** vena cava, **(E)** vena porta, and **(F)** aorta.

We first assessed whether various vessel types are suitable for isolation and culture. All used vessel types, the aorta, vena cava, and vena porta, were found suitable for the inversion and suturing steps necessary for EC isolation. We primarily used vessels with an approximate length of 5 cm, but the technique was also applicable for vessels as short as 1 cm (Oosterhoff et al., 2016). Canine VSMCs and CaPECs were derived from all used vessels. Cultures of VSMCs presented a “hill and valley” morphology when confluent within 3–7 days (Figure 1A–C). CaPECs became confluent within 1–2 weeks (Figure 1D–F), regularly displaying a cobblestone-like morphology. After the first confluency, the cells could be passaged weekly, 1:4 up to passage 8.

### Characterization

#### Whole Transcriptome Sequencing

**FIGURE 2:**
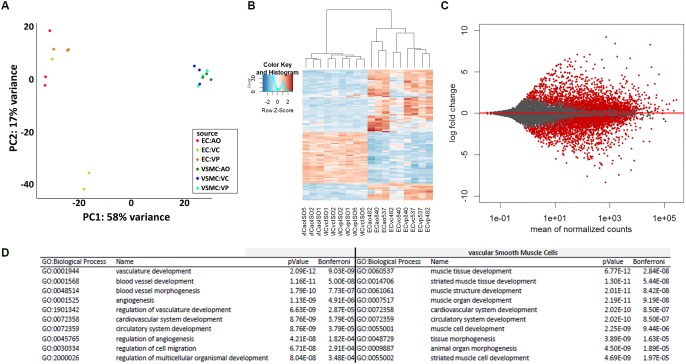
Differences in transcriptome profile between endothelial cells and vascular smooth muscle cells of different origins. Principal component analysis **(A)** and heatmap analysis **(B)** revealed a separate clustering of CaPECs compared to VSMCs. An MA-plot **(C)** depicts the high number of significantly differentially expressed genes when comparing the transcriptome of cells originating from the canine endothelial layer with cells originating from the smooth muscle cell layer. In total, 1936 genes (indicated as red dots) were differentially expressed with an adjusted *p*-value < 0.1. **(D)** Enrichment analysis based on Gene Ontology biological processes shows the top 10 activated processes in both cell types.

A principal component analysis of the mRNA levels in ECs and VSMCs distinguished the two cell types (Figure 2A), and a heatmap analysis of gene expression (Figure 2B). Transcriptome comparison demonstrated significantly differentially expressed genes, where every red dot resembles a significantly differentially expressed gene (Figure 2C). To assess the transcriptomic characteristics based on Gene Ontology, we selected the 100 most-expressed genes per cell type based on reads per kilobase million (RPKM). We detected enrichment of multiple vasculature-related biological processes in CaPECs and in contrast, detected processes related to muscle developmental in VSMCs, confirming the functional difference of the isolated cell types. The 10 most-activated processes per cell type are shown in Figure 2D.

**FIGURE 3:**
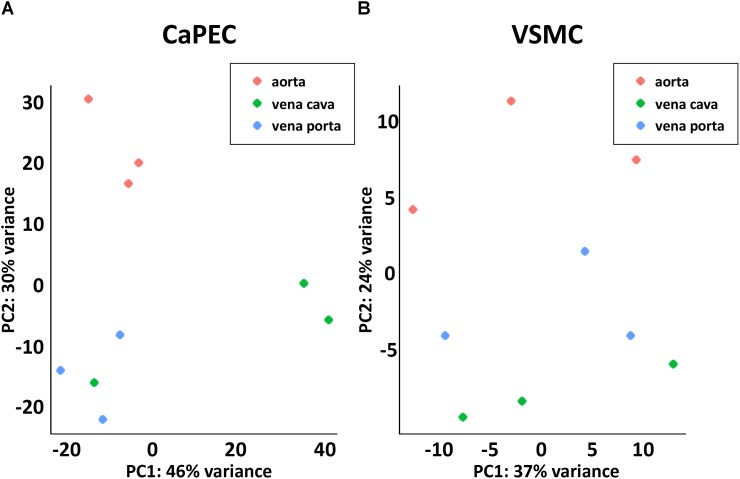
Transcriptome differences between cells derived from different blood vessels. **(A)** Principal component analysis revealed distinct clustering of CaPECs based on original vasculature. The samples were plotted in two dimensions using their projections onto the first two principal components. **(B)** Principal component analysis showed less prominent clustering depending on the vascular origin of VSMCs.

Furthermore, whole transcriptome data showed distinct differences between arterial and venous ECs (Figure 3A). We identified 508 genes that were differentially expressed (*p*-value < 0.05) when comparing CaPECs originating from the aorta to CaPECs originating from both the vena cava and vena porta combined. These genes exhibited log2fold changes ranging from −0.4 to −7.1 or between 0.4 and 9.5. These differences in vascular origin were less prominently demonstrated in VSMCs (Figure 3B); we identified 137 genes that were differentially expressed (*p*-value < 0.05) with maximum log2fold changes of less than −1.7 and more than 2.9, respectively.

#### Differentiation Markers

**FIGURE 4:**
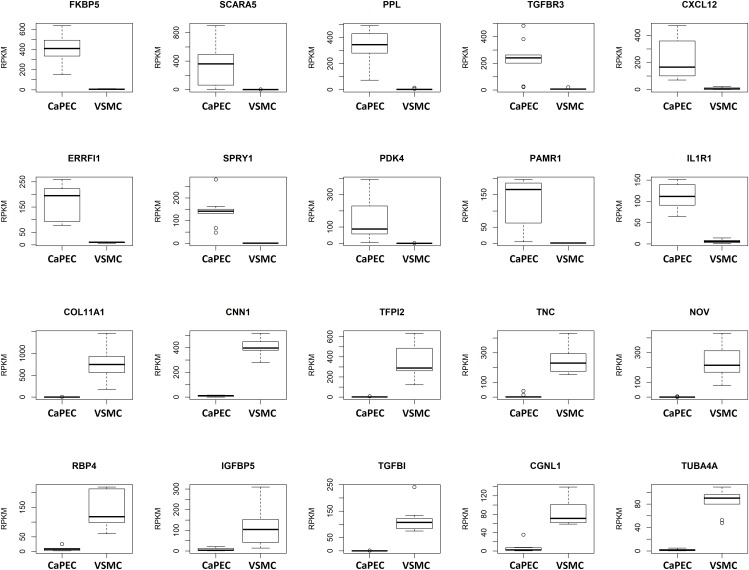
Differentiation markers for CaPECs and VSMCs. The 20 most significant differentially expressed genes between CaPECs and VSMCs based on DESeq2 analysis of the RNA sequencing data. Results are shown as reads per kilobase million (RPKM). The boxplots depict groups of numerical data through their quartiles per gene for the CaPECS (*n* = 9) and the VSMCs (*n* = 9). Genes with significantly higher expression in CaPECs in comparison with VSMCs were *FKBP5*, *SCARA5*, *PPL*, *TGFBR3*, *CXCL12*, *ERRFI1*, *SPRY1*, *PDK4*, *PAMR1*, and *IL1R1*. Genes expressed higher in VSMCs were *COLL11A1*, *CNN1*, *TFPI2*, *TNC*, *NOV*, *RBP4*, *IGFBP5*, *TFGBI*, *CGNL1*, and *TUBA4A*.

We further analyzed the transcriptome and identified differences in the top 10 genes when comparing CaPECs and VSMCs (Figure 4). The top 10 of genes specifically expressed in CaPECs were *FKBP5*, *SCARA5*, *PPL*, *TGFBR3*, *CXCL12*, *ERRFI1*, *SPRY1*, *PDK4*, *PAMR1*, and *IL1R1*; genes specifically expressed in VSMCs were *COL11A1*, *CNN1*, *TFPI2*, *TNC*, *NOV*, *RBP4*, *IGFBP5*, *TGFBI*, *CGNL1*, and *TUBA4A*. *P*-values ranged from 4.1^∗^10^−4^ to 4.1^∗^10^−5^.

**FIGURE 5:**
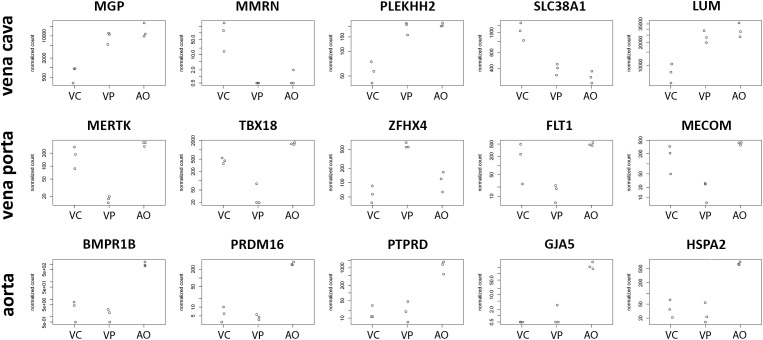
Differentiation markers in ECs from three different vessels. The five most significantly differentially expressed genes in CaPECs originating from the vena cava, vena porta, and aorta. In vena cava, *MGP*, *MMRN*, *PLEKH2*, *SLC38A1*, and *LUM* are specifically expressed, while in cells from the vena porta, *MERTK*, *TBX18*, *ZFHX4*, *FLT1*, and *MECOM* were expressed specifically. In CaPECs from the aorta, a specific expression pattern was found for *BMPR1B*, *PRDM16*, *PTPRD*, *GJA5*, and *HSPA2*. VC = vena cava, VP = vena porta, and AO = aorta.

CaPECs from the three vessel origins displayed specific expression patterns. The top five genes specifically expressed per sample group are depicted in Figure 5. For CaPECs from vena cava, these genes were *MMRN*, *PLEKH2*, *SLC38A1*, and *LUM*, and for vena porta, these were *MERTK*, *TBX18*, *ZFHX4*, *FLT1*, and *MECOM*. CaPECs originating from the aorta display a distinct expression patterns for *BMPR1B*, *PRDM16*, *PTPRD*, *GJA5*, and *HSPA2*.

#### Immunofluorescence Staining

**FIGURE 6:**
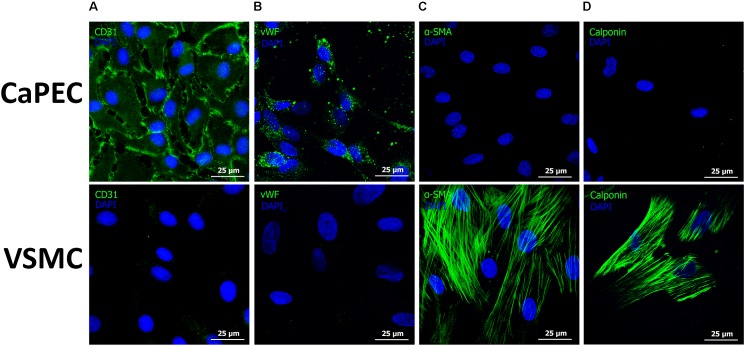
Immunofluorescent staining of markers for endothelial cells and vascular smooth muscle cells. Representative pictures of cells derived from the vena cava are shown. Membranous staining of endothelial cell marker CD31 **(A)** and cytoplasmic staining of vWF **(B)** were observed in CaPECs, while VSMCs did not show any positivity for these markers. Actin filaments were stained positive for both α-SMA **(C)** and Calponin **(D)** in VSMCs, but not in CaPECs.

Homogenous membranous staining of endothelial marker cluster of differentiation 31 (CD31) and von Willebrand factor (vWF) were found in CaPECs derived from the aorta, vena cava, and vena porta (Figure 6A,B), whereas VSMCs from the same vessels did not result in any positive staining. Mesenchymal marker alpha-smooth muscle actin (α-SMA) and vascular smooth muscle marker Calponin were stained positive in VSMCs, but not in the CaPECs (Figure 6C,D). Isotype controls did not show any staining (data not shown), indicating the specificity of the primary antibodies.

### Endothelial and Smooth Muscle Cell Functionality

#### 3D Spheroid Assay

**FIGURE 7:**
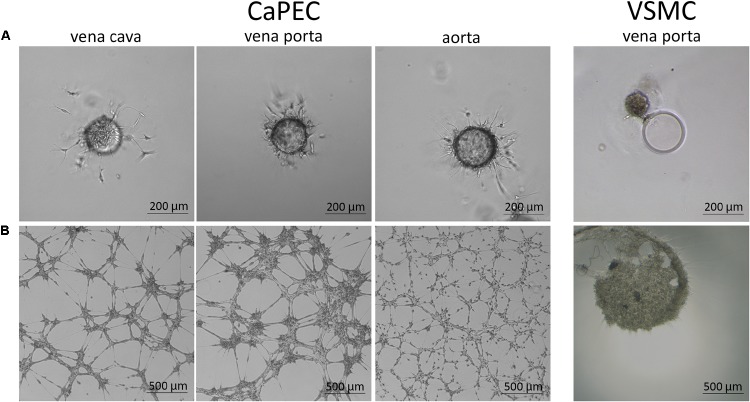
Angiogenic capacity of CaPECs and VSMCs. The functionality of CaPECs was assayed using two different methods. **(A)** 3D spheroid model. CaPECs derived from the vena cava, vena porta, and aorta showed sprouting capacity. VSMCs that were introduced in this assay showed no indication of sprouting capacity and formed a cluster. **(B)** All CaPEC cultures proved their angiogenic capacity in a tube formation assay. Networks of CaPECs originating from veins showed robust networks, whereas networks of arterial-derived endothelial cells appeared finer and more evenly spread. VSMCs originating from the same vessels did not show this network formation property.

Migration ability was studied by embedding the cells into a 3D spheroid model. CaPECs from all used blood vessels demonstrated branching sprouts originating from the spheroid bead; representative images are shown in Figure 7A. The VSMCs were not able to adhere to and distribute on the spheroid nor possessed the sprouting capability as observed in CaPECs. Instead, clustering was seen next to the beads.

#### Tube Formation Assay

All CaPEC cultures showed angiogenic capacity after 6 hours of incubation on IBIDI micro-slides. A morphological difference was observed between CaPECs derived from different vessel types (Figure 7B): angiogenesis in venous-derived CaPECs appeared as a robust network of cells, leaving relatively large areas of the matrix empty; and in contrast, arterial-derived CaPECs formed finer structures. This arteriovenous difference was detected in all three donors. VSMCs, on the other hand, did not show any network formation, and simply clustered together, confirming the functional difference between CaPECs and VSMCs.

#### Contraction Assay

**FIGURE 8:**
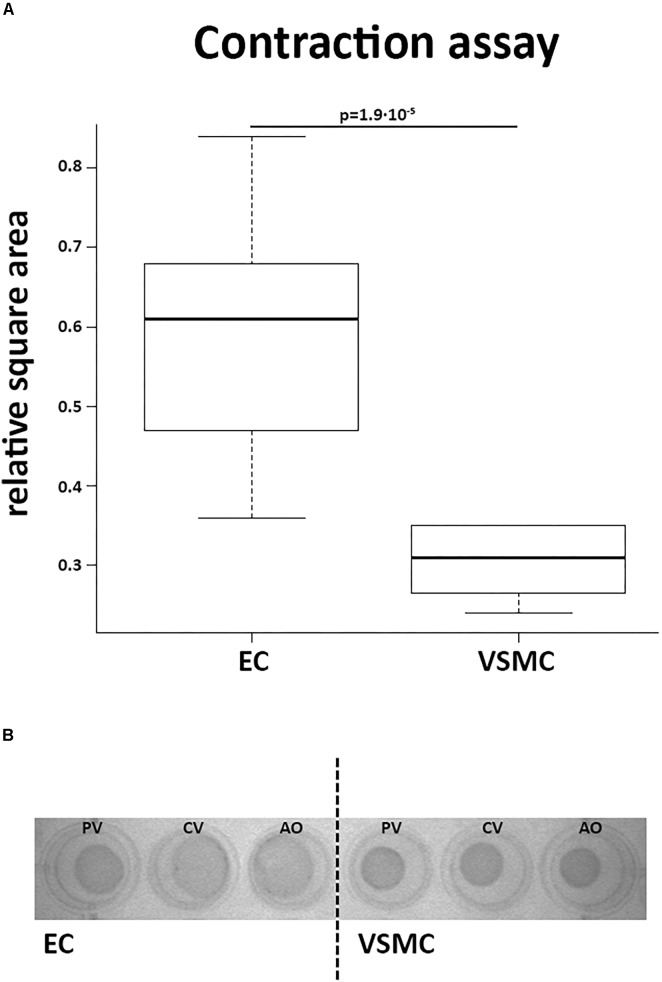
Contractile function of vascular smooth muscle cells. A collagen gel contraction assay revealed contractile function of VSMCs after 48 h of incubation. **(A)** VSMCs introduced into this assay showed more contraction of the collagen discs than CaPECs, determined by calculating the relative square area (*p* = 1.9^∗^10^−5^, non-parametric test). The boxplot depicts the square area per origin in quartiles for the CaPECS (*n* = 12) and the VSMCs (*n* = 12). **(B)** A representative collagen disc of both cell types derived from vena porta (PV), vena cava (CV), and aorta (AO).

Contractile function of VSMCs was studied in a collagen-based contraction assay. The square area of the discs was normalized to control discs without cells. VSMCs from all derived vessels showed significantly more contraction compared to CaPECs (*P* < 0.01, Figure 8A). A representative image of the discs at the time of measurement is shown in Figure 8B. In addition, in a separate experiment, we showed that ECs were viable on collagen discs and contractibility of the VSMCs was confirmed by adding skin and lung fibroblasts from dogs (Supplementary Figure S1).

## Discussion

The increasing incidence of cardiovascular disorders demands improved *in vitro* research models that better mimic native *in vivo* conditions in human patients. The interaction of ECs with their supporting cells (e.g., VSMCs) is indispensable for (micro)vascular development and ECM composition (Korff et al., 2001; Jain, 2003) and must be considered when translating this to a cell culture model. Indeed, earlier findings have reported the importance of VEGF secreted by VSMCs for the *in vitro* migration ability of HUVECs (Heiss et al., 2015). Unfortunately, these complex interactions are not recapitulated in current HUVEC models. Mouse models too have proven valuable for studying human diseases but are genetically engineered and due to differences in physiology, genetic models might not fully represent naturally occurring disorders caused by patency of embryonal vessels (Segi et al., 1998; Loftin et al., 2001; Eleftheriou et al., 2016). Therefore, it is essential to choose an animal model where cardiovascular diseases naturally occur. Blood vessels in the canine are more similar in size and function to human vessels, and also reduce the need for genetic manipulation (Oosterhoff et al., 2016; van Steenbeek et al., 2016). Additionally, the existing overlap in commonly occurring diseases in both human and dogs such as patent ductus arteriosus and vascular stenosis is of great benefit when cells originating from canine vessels are used in disease modeling (Gittenberger-de Groot et al., 1985; den Toom et al., 2016; Laborda-Vidal et al., 2016; Van den Bossche and van Steenbeek, 2016). The novel vessel inversion technique we describe allowed us to obtain viable CaPECs and VSMCs from vessels as small as 1 cm in length (Oosterhoff et al., 2016). In addition, the isolated cells could be directly cultured, negating the need for FACS sorting. We anticipate that this method can be easily translated to other species.

We describe the isolation and characterization of CaPECs and VSMCs from the same blood vessels (the aorta, vena cava, and vena porta) of three adult dogs and provide a novel comparison of these cell types. As expected, CaPECs and VSMCs differed in morphology, transcriptomic landscape, expression of cell type specific markers, and functionality. More specifically, mRNA expression of several genes differed significantly between CaPECs and VSMCs. The top 10 highest expressed genes of both cell types revealed cell type-specific genes. Besides *TGFBR3* and *CXCL12*, which play a role in response to hypoxia, the list of genes with specific expression in ECs does not display an enrichment for functions clearly correlated with behavior of ECs. The genes specifically expressed in VSMCs are mostly correlated to cytoskeleton composition: TGFB1, COL11A1, NOV, TNC, and TFPI2 are known ECM proteins, TUBA4 is involved in cytoskeleton organization, whereas CGNL1 plays a role in actin filament organization (Ashburner et al., 2000). Further investigation is required to confirm whether these proteins specifically define cell origin.

The presence of the endothelial markers *CXCL12*, *TGFBR3*, *FKBP5*, and *ERRFI1* could be confirmed in other species based prominent expression in publicly available whole transcriptome sequencing data [mouse ECs in GSE114805 (Natarelli et al., 2018) and GSE104530 (Nelson et al., 2018) and HUVECs in GSE76743 (Voellenkle et al., 2016)]. Remarkable is the expression of *TNC* in murine aortic ECs (Natarelli et al., 2018). The fact that TNC is commonly known as ECM component and *TNC* is not expressed in ECs (Radwanska et al., 2017) indicates a possible contamination in the reported study. Furthermore, expression of *TFPI2* found in the human umbilical cord ECs (Voellenkle et al., 2016) is in agreement with previous findings (Beeravolu et al., 2016), whereas expression was not detected in the adult CaPECs. This specific expression in extraembryonic tissue (Udagawa et al., 1998) stresses the additive value of adult ECs in disease modeling. CaPECs isolated from the aorta or venous vessels displayed behavioral and morphological differences in the spheroid and angiogenesis assays; this arteriovenous differences in expression based on anatomical origin was greater in CaPECs compared to VSMCs. Additionally, principal component analysis revealed a marked difference between CaPECs derived from various anatomical locations, emphasizing the importance of studying endothelial function in representative EC populations.

Isolating ECs and VSMCs and culturing these cells in 2D will have its effect on transcriptome and function compared to cells in their natural environment. Response to loss of mechanical, chemical, and biological cues forces them from a quiescent into a proliferative state which might negatively influence the resemblance to its *in vivo* counterpart. Current advances in single-cell analysis defined transcriptional differences already due to serum (Guo et al., 2016). However, even when adjusting for the effect of serum by replacing it with human supplements, still *in vitro* expansion in itself results in increased transcriptome diversity (Kim et al., 2018). Although the isolation and expansion of CaPECs and canine primary VSMCs in its current state will not completely reflect their *in vivo* physiology, from a fundamental perspective, these cells are potentially very valuable. A limiting factor during this study was the *n*-value of donor tissues. However, gene expression profiles of both arterial- and venous-derived ECs confirm the differences between subtypes observed in previous studies (Aird, 2007; Aranguren et al., 2013). Moreover, we observed a functional difference in angiogenic capacity between ECs from different vessels: refined networks in ECs derived from the aorta and more robust networks when derived from the vena cava and vena porta.

The use of ECs and VSMCs from the same vessel of interest in a co-culture model may provide insight into cell behavior in both healthy and diseased circumstances. In addition, primary cell cultures create new possibilities to study (congenital) cardiovascular diseases, and can be incorporated into 3D *in vitro* bioreactors and microfluidic devices, that better mimic natural vasculogenesis, cell behavior and (dys)function in diseased vessels. For example, cells originating from vessels present after patency of embryonal vessels like the patent ductus arteriosus or patent ductus venosus can be cultured and used for disease modeling research (den Toom et al., 2016; Van den Bossche and van Steenbeek, 2016).

## Author Contributions

The authors all contributed significantly to and carefully read this manuscript. LO performed the experiments and drafted the manuscript. HK and TS performed the isolation of the endothelial cells. MvW, JdG, TV, and FZ performed the experiments. BvB and JF conceived and designed the experiments. MM, NvdD, and NL performed the experiments and conducted bioinformatic analysis. LP drafted and edited the manuscript. BS and FvS together conceived the study, designed the experiments, analyzed the data, and drafted the manuscript.

## Conflict of Interest Statement

The authors declare that the research was conducted in the absence of any commercial or financial relationships that could be construed as a potential conflict of interest.

## Supplementary Material

FIGURE S1 Viability of endothelial cells in collagen discs. **(A)** Contraction assays performed on CaPECs, VSMCs, skin fibroblasts and lung finroblasts show lack of contraction in CaPECs compared to the other cell types. **(B)** Trypan Blue staining of cells in collagen discs confirmed that all cell types were still viable after 48 hours.
