# Supplementary figures and images for: Characterization of Endothelial and Smooth Muscle Cells From Different Canine Vessels
Source: Front Physiol. 2019 Feb 12;10:101. doi: 10.3389/fphys.2019.00101 (PMC6379353; doi:10.3389/fphys.2019.00101)

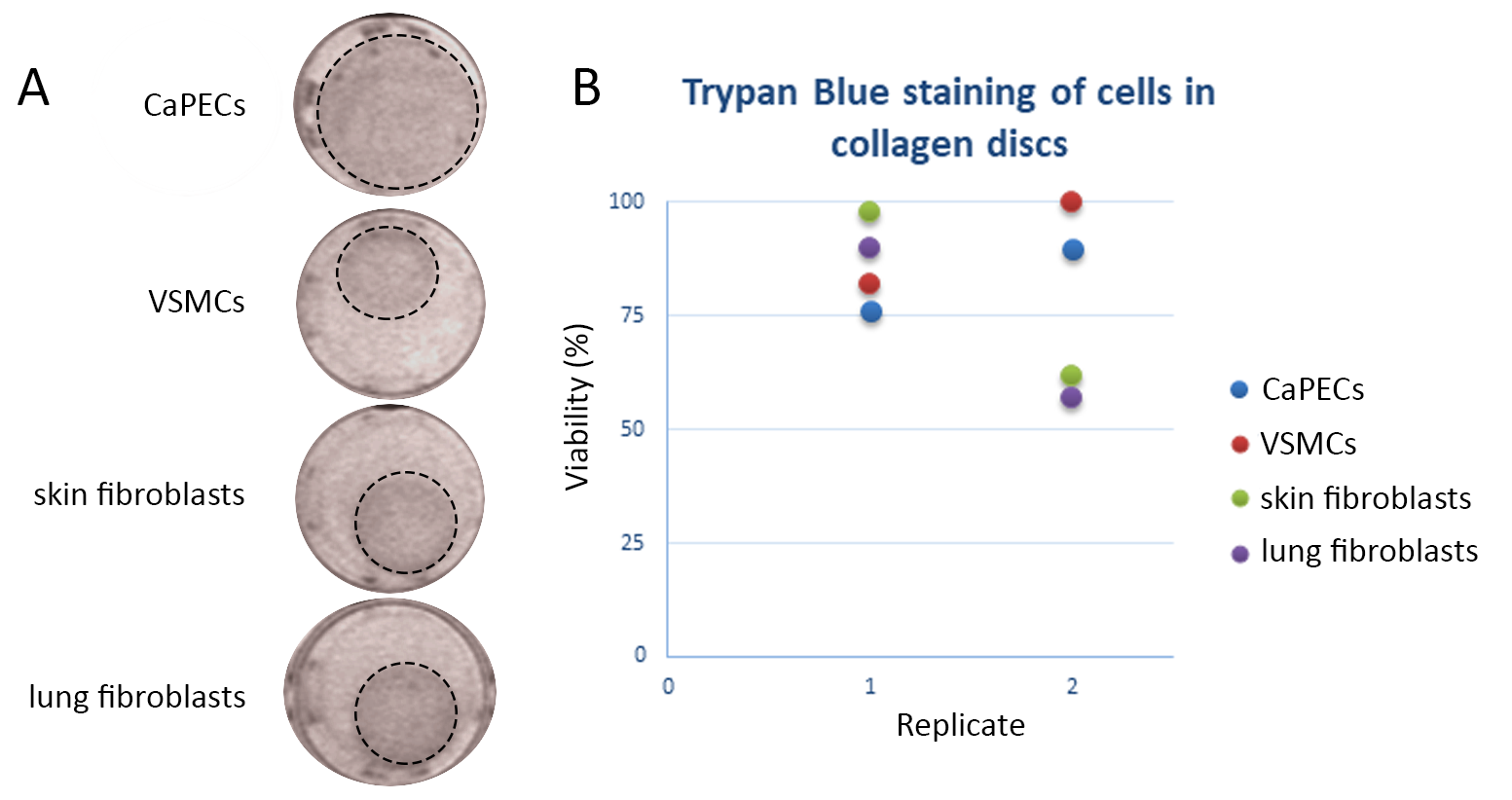

Supplement: FIGURE S1 — Viability of endothelial cells in collagen discs. (A) Contraction assays performed on CaPECs, VSMCs, skin fibroblasts and lung finroblasts show lack of contraction in CaPECs compared to the other cell types. (B) Trypan Blue staining of cells in collagen discs confirmed that all cell types were still viable after 48 hours. [file Image_1.TIF]
